# Analysing genome sequences and associated metadata during the COVID-19 pandemic in Iraq revealed points to be improved: An observational retrospective study

**DOI:** 10.1371/journal.pone.0326750

**Published:** 2025-06-30

**Authors:** Ali Hadi Abbas, Aoula Al-Zebeeby, Mohammed Al-Saadi, Ahmed Jasim Neamah

**Affiliations:** 1 Department of Microbiology, Faculty of Veterinary Medicine, University of Kufa, Al-Najaf, Iraq; 2 Department of Pathology, Faculty of Veterinary Medicine, University of Kufa, Al-Najaf, Iraq; 3 Department of Internal and Preventive Medicine, College of Veterinary Medicine, University of Al-Qadisiyah, Al-Qadisiyah, Iraq; 4 Department of Microbiology, College of Veterinary Medicine, University of Al-Qadisiyah, Al-Qadisiyah, Iraq; Al Muthanna University, IRAQ

## Abstract

The COVID-19 pandemic started in Wuhan China and rapidly transmitted worldwide, the illness is characterised by respiratory manifestations like coughing, breathing difficulties and pneumonia that could lead to death. Real-time whole genome sequencing of severe acute respiratory syndrome corona virus 2 (SARS-CoV-2) was adopted in many countries to track the infection dynamics and evolution of the virus. In parallel with the global efforts, genome sequencing trials were established in Iraq during the COVID-19 pandemic, however, this new approach has not been assessed yet. Therefore, for better readiness and improvement for future pandemics, here we obtained all genomes of SARS-CoV-2 virus from Iraq (182) that were deposited in National Center for Biotechnology Information (NCBI) during the period (2020–2023). Statistical analyses of sample size, distribution and other epidemiological parameters from associated metadata, as well as the quality of genome sequences were assessed. Our data analyses highlighted some drawbacks that could be improved, namely, that most genomic sequences (62%) were collected from only two cities, a low sample size was noticed and sequencing quality was inconsistent. There was a shortage and impairment of sequencing facilities especially those of the Ministry of Health. Consequently, genome sequencing should be achieved in centres that produce the best quality. The results revealed the importance of well-documented and high-quality sequences that represent many important cities in the country, which is crucial to draw a clear projection for health officials on infection dynamics and tracking viral evolution to help in taking successful steps towards infection control.

## Introduction

COVID-19 defined as an acute respiratory disease caused by a novel coronavirus of Severe Acute Respiratory Syndrome Coronavirus 2 (SARS-CoV-2). This illness was first detected in Wuhan Province, China [1,2].

The latest update from WHO on COVID-19 global cases until 27th April 2025, there are over 777,745,434 detected cases from 240 countries and more than 7 million deaths [3]. When infected individuals cough, sneeze, talking, or breathe, tinny droplets will be generated containing the virus, which will be discharged into the surrounding air. Additionally, the virus can be transmitted from person to person via direct contact or indirect contamination of shared objects [4]. The clinical signs of the illness vary from mild or even no symptoms to a fatal condition [5]. The most predominant clinical symptoms are fever, headache, coughing, difficulty breathing, shortness of breath, diarrhoea, nausea, vomiting, chills, body aches, sore throat, congestion/runny nose, and loss of smell or taste. Ultimately, the infection could lead to pneumonia or respiratory failure, septic shock, heart attacks, liver problems, and death.

At the onset of the newly emerged disease, in Wuhan, China in late 2019, the causative agent was not yet known. The first genome sequence was generated in January 2020 from bronchial lavage. This genome sequence revealed a *coronaviridae* virus belongs to the genus Betacoronavirus [6], with a genome sized ~ 30 kb of single-stranded positive sense RNA, containing five Open Reading Frames (ORFs) encoding viral proteins named as: RNA-dependent RNA polymerase ORF1a and b, spike protein (S), envelope (E), membrane (M) and an ORF for the nucleocapsid (N) [6,7].

COVID-19 pandemic showed high vulnerability of health systems worldwide, especially in the low and middle-income countries, some of these issues are lack of Personal Protective Equipment (PPE), testing kits, inadequate transport chains, trained individuals, lack of enough funding to cope with a pandemic at such scale [8,9].

Right after the start of the pandemic, real-time whole genome sequencing consortiums like the first COVID-19 Genomic UK Consortium (COG-UK) started in March 2020, an integrated national scale of the SARS-CoV-2 genomic surveillance network were established in different countries to track viral evolution and mutations lead to increase pathogenicity or transmissibility [10]. Tracking viral evolution is essential as viruses are constantly evolving and changing pathogenicity to hosts [11,12].

It has been proven that whole-genome sequencing (WGS) is the most precise and accurate tool to follow up on the transmission dynamics of epidemics and give the required information for the outbreak control to decision makers [13]. Real-time WGS was applied during the emergence of the West African Ebola outbreak between 2014–2016 [14,15]. Since then, real-time analysis and sharing of sequencing data during outbreaks has become routine in public health assets as a fundamental measure of outbreak response [14]. However, inconsistent efforts and incomplete metadata with lack of important parameters have a huge impact on downstream data analyses [16,17].

During the COVID-19 pandemic new approaches were developed to track infection and monitoring newly emerged variants this was through test waste water for SARS-CoV-2. This strategy offered fast and affordable choice to track emergence and evolution of the virus as it easy to get access to the samples and proven successful [18,19].

In Iraq, the first genome sequence of SARS-CoV-2 was released in 2020 [20], followed by other attempts to sequence COVID-19 cases in certain regions of the country to detect possible emerging lineages such as alpha variant and delta variant [21,22]. In this study, an observational retrospective study was adopted, we analysed the genomic sequences and linked metadata deposited in NCBI public repositories in order to assess the sequencing efforts for tracking SARS-CoV-2 virus in Iraq and to inform health policy makers to improve practices for future outbreaks. The achieved analyses revealed aspects to be improved in order to upgrade this capability in Iraq.

## Methods

### SARS-CoV-2 genome sequences

The genome sequences used in this study were obtained from the NCBI website that dedicated to the SARS-CoV-2 data hub (https://www.ncbi.nlm.nih.gov/labs/virus/vssi/#/virus?SeqType_s=Nucleotide&Country_s=Iraq&HostLineage_ss=Homo%20sapiens%20(human),%20taxid:9606&SLen_i=27000%20TO%2030000&CreateDate_dt=2020-04-28T00:00:00.00Z%20TO%202023-09-06T23:59:59.00Z), the following search filtering was applied; sequence length were set to (27000–30000), the option of release date was set from (April 2020–7^th^ September 2023) and Iraq was selected for geographical region option, all other options were left on default settings. This search involved whole genome sequences from Iraq covering the time from the start of the pandemic cases in Iraq, i.e., (April 2020–7^th^ September 2023). A multi-FASTA file of 182 whole genome sequences was downloaded (see S1 Table), and a metadata file contains all listed information about these samples is provided (see S2 Table).

All data analyses were achieved using the R platform via RStudio [23]. The complete R code used in this study is provided in (S1 File).

### Geographical distribution of genomes across Iraq map

Special R packages implemented in the RStudio interface version (2023.09.1 + 494) [23].

### SARS-CoV-2 variants assignment

Genome sequences of the SARS-CoV-2 virus were assigned to the variants by pangolin software [24] for variant detection, and they are included in the metadata file downloaded from NCBI (S2 Table).

### Statistical analyses and plot generation

All statistical analyses were performed on R platform version 4.3.2 (2023-10-31) [25]. The Kruskal-Walis test of one-way ANOVA for skewed data implemented in “rstatix” package [26] were applied to test if there were significant differences between the analysed groups of genomes. A Pairwise Wilcox test within the “rstatix” package was achieved to detect which group had a significant statistical difference from other groups.

Logarithm base 10 and square root normalisation were applied to achieve more representative data visualisation plots using R version 4.3.2. In addition to several R packages used in generating plots were listed in (S3 Table).

## Results

We analysed these genomic sequences along with the provided metadata collected from samples of COVID-19 cases from Iraq. It is worth mentioning that many aspects are missing from the metadata linked to this genomic dataset, for example gender, age, occupation, travel status, nationality, underlying diseases, and purpose of study.

### Distribution of genomes from Iraq

**Fig 1:**
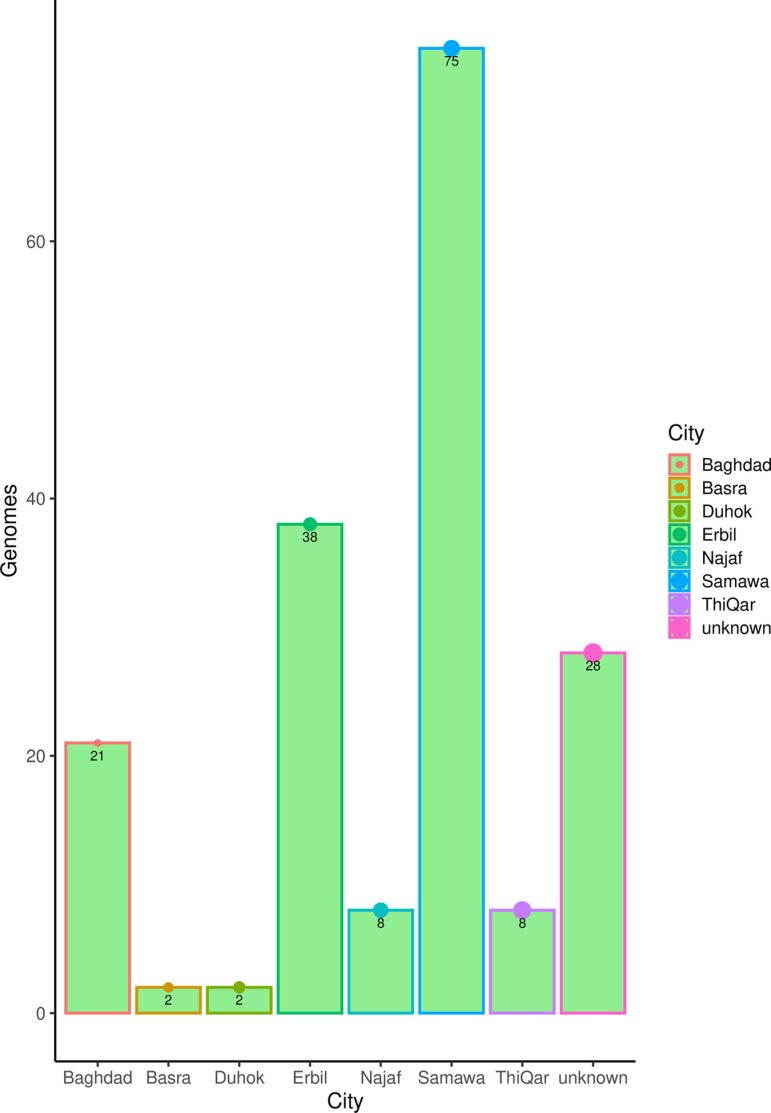
Distribution of SARS-CoV-2 genomes collected from Iraq according to the NCBI repositories. A bar plot represents the actual number of genomes collected from each city; “unknown” refers to genomes from Iraq with no location assigned. The plot generated using the R platform, and the package ggplot2.

A total of 182 whole genome sequences of SARS-CoV-2 were taken from cases around the country from the time given (i.e., from April 2020- September 2023), in comparison to WGS sequences from developed countries such as the UK and USA were 3, 218, 629 and 3,403, 246, respectively using the same research criteria in the NCBI database. This reveals a huge difference in the number of WGS of SARS-CoV-2 between Iraq and pioneered countries. Iraq these genomes were collected from seven different Iraqi cities, and 28 (15.4%) genomes were taken from unspecified locations in Iraq. Most samples were sequenced from one city (Samawa) with 75 (41.2%) genomes, followed by Erbil 38 (20.9%) followed by the capital city of Iraq (Baghdad), and the rest four cities were represented by a very small number of genomes (Fig 1).

### Assessment of genome quality

Genome quality evaluation is an important step for ensuring the quality of genomic data and its liability for further analyses. The completeness of the genome is crucial for perfection of comparative genomics and whole genome SNP mapping, which consequently affects the final variant assignment of SARS-CoV-2. The presence of gaps in the genome sequences reduces the efficacy of the a forementioned analyses. Usually, gaps in the genome are missing DNA bases, which are represented by the “N” character instead of normal DNA characters (“A”, “C”, “T”, and “G”).

**Fig 2:**
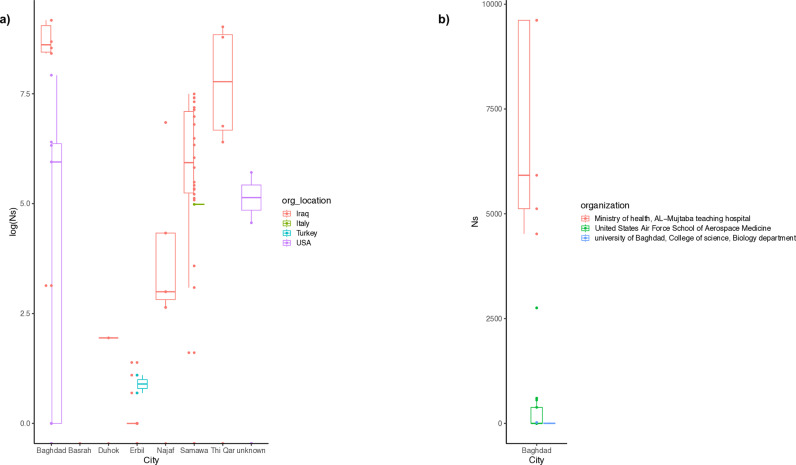
Genome quality assessment. **A)** Box plot of genome quality assessment was analysed according to the number of ambiguous bases in the genome sequence represented by the character “N”. The Y axis represents the logarithmic normalisation of the numbers of “N” characters in the genomes from each city. Coloured by the country of the laboratory where the sequencing was achieved. **B)** The same as in A but focused only on genomes from Baghdad, which showed the highest number of ambiguous bases; Y axis showed the actual number of Ns. Box plots are coloured according to the organisation of the sequencing laboratory. Plots were generated using the R statistical platform and library ggplot2.

We calculated the number of “N” characters in genomes collected from each assigned sequencing institutions or location as recorded in NCBI meta data. Analysis of variance showed that genomes from Baghdad, followed by ThiQar, have a significant increase in gaps (p < 0.05) in comparison to all other locations. Further analysis for gnomes from Baghdad that were collected or sequenced by 3 different institutions revealed that genomes from the Iraqi Ministry of Health had a significant level of gaps (Fig 2B), p*-*value < 0.0001.

The genomes with the best sequence quality were those from Erbil and Duhok with the least number of “Ns”, while the highest number of gaps can be noticed in genomes sequenced from Baghdad and from ThiQar (Fig 2A).

### SARS-CoV-2 genomes across time and location

**Fig 3:**
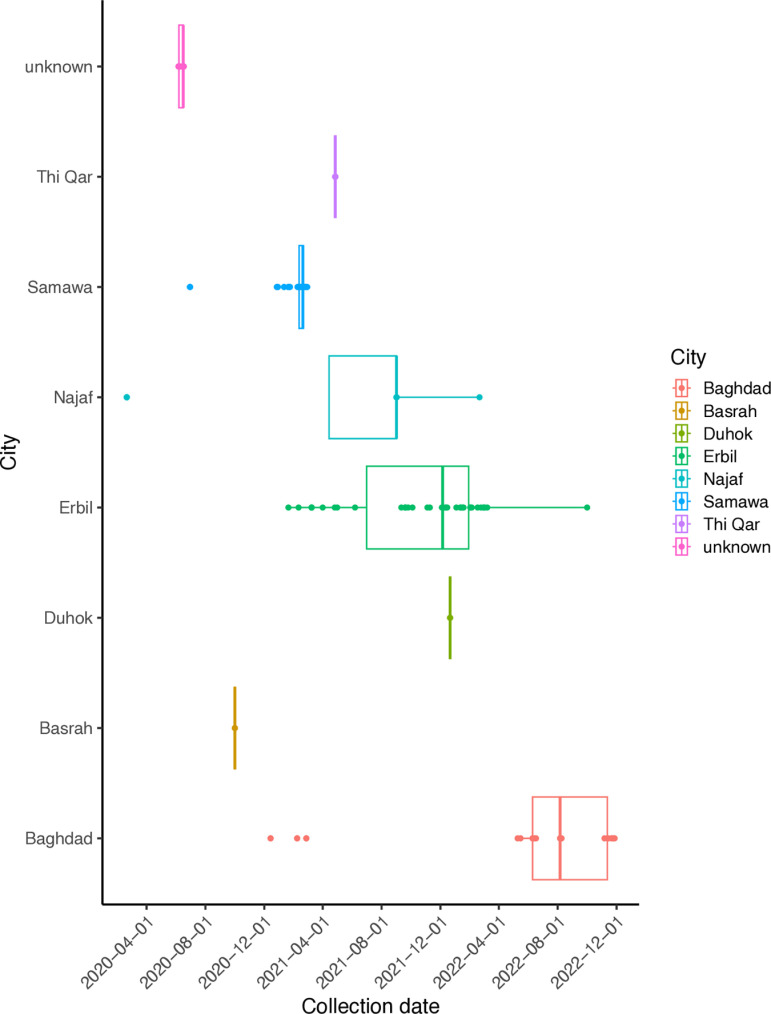
Distribution of genomes from the NCBI database from Iraqi cities across time. This plot represents the collection date of genomes (X axis) as a timeline for each city (Y axis), colour-coded according to the sample location.

The collection dates covered the time from the start of COVID-19 cases in Iraq in April 2020 to the first of December 2022, the samples were collected unevenly (Fig 3). The broader time coverage was noticed in samples collected from Erbil as it covered the period from December 2020 to October 2022 followed by samples from Baghdad and Najaf; however, the collection date of samples from Baghdad showed biases towards the second half of 2022, while samples from Najaf were only 8 samples that were collected sporadically, mainly during year the 2021. Significantly, samples from Samawa were confined within a relatively narrow timeframe, which was in the year 2021. The rest of the samples from an unspecified location, ThiQar, Duhok and Basrah were collected at a specific time (Fig 3).

### SARS-CoV-2 variants across location and time

**Fig 4:**
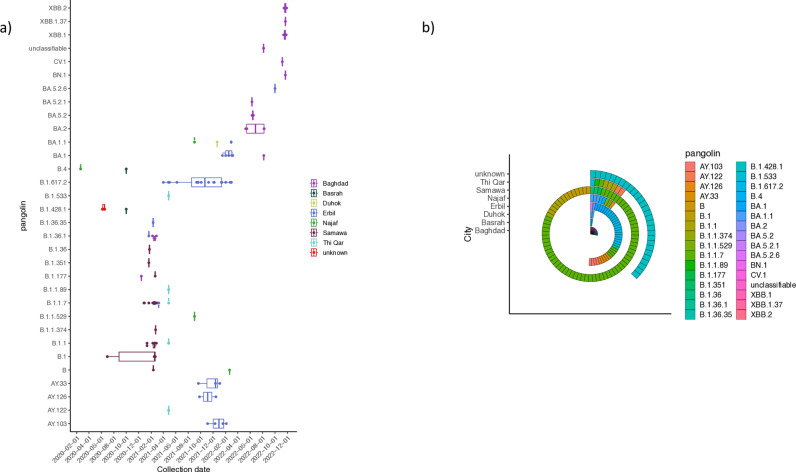
Shows the distribution of variants across time and location. **A)** The Y axis represents the variants of SARS-CoV-2 according to pangolin classification. Points were coloured according to the location of genomes (cases). Plots were generated by the R platform and library ggplot2. **B)** Variants that were isolated from each city are coloured according to pangolin classification.

According to the genomic data, the SARS-CoV-2 variants were plotted against time and location (Fig 4). Remarkably, samples isolated from patients in Baghdad were represented by a broader number of variants (11 variants) most variants were the BAx variants and the newly emerged hybrid variant XBBx, which were positioned in the latest collection dates. While samples from Erbil were represented by 10 variants, the B.1.617.2 lineage was the most lasting one over time; others were clustered within AYx variants and BA.1x variants. Although samples from Samawa formed the majority of genomes from Iraq, they only fall into 8 variants of Bx lineages. Notably, 28 samples from an unknown location in Iraq fall into only one variant B.1.428.1.

## Discussion

Globally, COVID-19 pandemic was the first to apply such an unpresented large-scale real-time genomic sequencing approach worldwide, which allowed researchers and health care policymakers both locally and internationally to draw plans for disease transmission, control and national and international preparedness by unravelling disease transmission dynamics and SARS-CoV-2 virus evolution, and the emerging of new variants in a real-time manner [27,28]. In Iraq, the first and only application of relatively large-scale whole genome sequencing on infectious agents was on the biggening of COVID-19 pandemic in a determination to meet global efforts to track SARS-CoV-2 virus infection and evolution. It is an important first step to use such an approach to provide valuable information during outbreaks to inform official health care entities [29]. External quality assessment of whole genome sequencing projects is essential to ensure better quality and improvement for future challenges [30,31].

In Iraq, we noticed that the genomic sequencing efforts are not centralised or nationally organised, it rather depends on individual research driven trials. In this study, we assess this new experience in Iraq to improve the overall process, increase awareness among authorities and health care specialists on points to improve, avoid misleading information and elevate the quality of the produced genomic data to meet global efforts on controlling COVID-19 pandemic and future outbreaks.

Most of the analyses in this study relied on state-of- the-art data visualisation software, that simplified and summarise huge and complex data in clear pinpoint figures. The use of illustrations and informative figures is highly recommended in the studies related to public health, to show global public health information as it simplified complex data, enhances communication, make it readable by non-technical individuals, easy to be viewed and transferred, as well as, showing trends in the data [32,33].

The associated meta-data of the genomic data set deposited in NCBI repositories showed that the whole genomic sequencing effort was not centrally organised, as different institutions (academic, health care officials, local and international organisations) were involved in sample collection, from a few locations and not evenly distributed across Iraq. Sample size and distribution are important factors that affect whether WGS studies are epidemiologically informative [34]. Although some locations, like Erbil, showed the best distribution of sample collection across time, the overall trend clearly presents an inconsistent timeframe for the sample collection date, which might reflect an unclear view of the evolution and dynamics of the pandemic [35]. Real-time sample collection and sequencing can facilitate not only evolution dynamics but also reveal the potential source of infection, portal of entry, location of the first emerging of a given SARS-CoV-2 variant, which has a big impact on how to choose and apply effective control measures that could help halt the transmission, to the resolution of a given outbreak even within a given health setting [16]. In this study, the distribution of SARS-CoV-2 variants across locations revealed a different behaviour regardless of the number of collected samples, with Baghdad having the highest number of variants, followed by Erbil and Samawa, respectively. These differences might be evoked due to the social specifications of each city: Baghdad as the capital of Iraq, which hosts the main international airport in the country, and Erbil, with similar specifications as the capitol of KRG (Kurdistan Regional Government), even though the number of genomes is much less than those from Samawa, this might suggest increasing the sampling and sequencing from these cities and other cities like Najaf, Basrah and Karbala as the latest cities have also been considered portal of entry to the country and places for annual mass gatherings for both national and international visitors for big religious events. The socio-spatial structure and people movement across cities or communities have major effect on disease transmission and causative agent evolution [36], population density, and geographical and environmental differences have an effect on disease dissemination [37]. The only location in Iraq that showed a unified variant (B.1.428.1) prevailed in a location that has not been specified (termed “unknown” in this study). Analyses of SARS-CoV-2 variant assignment and the sample collection time frame suggested a local, confined outbreak in this undefined location.

At the time of this study, the most recent variant identified was XBB variants, from samples isolated from cases in Baghdad. This is probably because Baghdad is the only location that has samples collected from the time of this variant emergence.

Genome quality is one of the most important parameters for a whole genomic study. The assessment of the quality of genomes from each location or institution was done by calculating the number of sequence gaps. The analysis suggests that the best complete genomes were those from the Kurdistan region (represented by genomes from Erbil and Duhok), while the worst case was the genomes from Baghdad. Further investigation on genomes from this city revealed that three sequencing institutions were involved in the generation of whole genome sequences. These institutions were the Ministry of Health, University of Baghdad, and US air force school of medicine. This analysis revealed that the main source of bad-quality genomes was those sequenced or processed in the Ministry of Health facility. This showed a major issue in the procedure of generating complete genome sequences from an important health institution. Hence, further training or scrutiny is needed over this facility on equipment and personal, to avoid the generation of low-quality genomes in future outbreaks or relying on better performing sequencing capacities like the ones in the KRG region.

We also noticed that there is many information missing from the metadata linked to this dataset, for example, age, gender, travel status, occupation, nationality, underlying diseases, and purpose of study (i.e., reasons beyond sampling). Incomplete metadata has a major effect on data-driven analyses [38]. It is worth mentioning that linking this metadata to the genome sequences in open-source global repositories has a major benefit to downstream data analyses and best possible piece of information [34]. Many metadata policies that can be adopted were proposed [39]. It is important to indicate that the result of this study is largely affected by the database resources and the metadata policy adapted by this data repository. Missing essential information such as temporal, demographic, geographical, social and even environmental metadata hindering the usability of shared WGSs, which reducing the benefits from data analyses to gain more informative inferences that help public health authorities to build a comprehensive picture of an outbreak [28,40,41].

In this study many issues were extrapolated from the available stored sequences in open access repository, most of these issues can be fixed to elevate the future work. This emphasizes on the importance of sharing pathogen sequence data and a complete set of metadata. While it highlighted common drawbacks like the lack of infrastructure, lack of experienced individuals, load on health sector, shortage in funding and cross contamination from different laboratories [8,42], it can also add up to mentioned previous issues in low and middle-income countries.

Some of noticed issues came from unparalleled centralised efforts with impaired funding, lack of training and lack of infrastructure hindered such nation-wide efforts, such issues were noticed in a number of low- income countries [43,44].

This study has relied on an open access repository provided by GenBank that stores genomes from all courtiers including Iraq, while this is a free to download, analyse and populate data, it is limited to what has been deposited in this datahub.

## Conclusion

The analyses highlighted many issues that could be largely improved. Specifically, sampling issues in the context of time and location, the lack of the appropriate patient’s records and the importance of adapting a unified effort to give a better outcome to the real-time-whole genome sequencing efforts. This analysis provides important information to the health policy makers in the country on how to resolve these issues to improve health practices to confront potential future outbreaks. We largely recommend devoting more fund and resource on genomic sequencing facilities to upgrade it and to deal with the pandemic pressure with a centralised programme to unit all efforts nationwide.

## Supporting information

S1 Table Whole genome sequences of 182 COVID-19 samples and corresponding accession numbers that were used in this study. (CSV)

S2 Table A metadata information has all listed information about the samples as it stored in GenBank. (CSV)

S3 Table R packages with corresponding references used for analysing data in this study. (XLSX)

S1 File The complete R code was used in this study to generate figures and to do statistical analysis. (TXT)
